# *In vivo* inhibition of TDO2 in fibroids results in widespread alteration in the tumor transcriptome

**DOI:** 10.1042/CS20260395

**Published:** 2026-05-26

**Authors:** Tsai-Der Chuang, Abigail Wiseman, Gabriela Alfaro, Sayna Pejouhesh Jahromi, Sepideh Pejouhesh Jahromi, Daniel Baghdasarian, Omid Khorram

**Affiliations:** 1 The Lundquist Institute for Biomedical Innovation, Torrance, CA, U.S.A.; 2 Department of Obstetrics and Gynecology, Harbor-UCLA Medical Center, Torrance, CA, U.S.A.; 3 Department of Obstetrics and Gynecology, David Geffen School of Medicine at University of California, Los Angeles, Los Angeles, CA, U.S.A.

**Keywords:** Fibroid, lncRNA, miRNA, TDO2, Xenograft

## Abstract

The present study aimed to characterize large and small RNA transcriptomic changes in fibroid xenografts from mice treated with the TDO2 inhibitor 680C91 for 2 months and to validate selected findings using qRT-PCR, protein analyses, and *in vitro* fibroid explant models. Large RNA next-generation sequencing revealed that 680C91 induced broad transcriptomic alterations, with enrichment of pathways related to the extracellular space, RNA processing, PI3K/AKT signaling, and calcium signaling. Small RNA sequencing identified enrichment of pathways associated with PI3K/AKT signaling, proteoglycans in cancer, and interleukin signaling. Key differentially expressed genes were validated in xenografts and fibroid explants. Treatment with 680C91 significantly reduced the mRNA expression of VDR, MMP11, MMP14, COL11A1, CBX4, LINC02568, LINC01310, LINC02544, and LINC02182, while increasing miR-584-5p expression. These changes were consistently observed in fibroid explants treated with 680C91 for 48 h. Corresponding decreases in protein levels of COL11A1, VDR, CBX4, MMP11, and MMP14 were also detected. Additionally, 680C91 inhibited AKT phosphorylation and reduced α-smooth muscle actin and vimentin expression. Importantly, all validated transcripts displayed expression patterns opposite to those observed in fibroid tissues compared with matched myometrium, with more pronounced effects in MED12-mutated tumors. These preclinical findings support TDO2 inhibition as a potential therapeutic strategy for uterine fibroids.

## Introduction

Uterine fibroids are common benign gynecologic tumors that affect a large proportion of women of reproductive age. These estrogen- and progesterone-dependent tumors are associated with significant morbidity, including abnormal uterine bleeding, pelvic pain, and infertility [1,2]. Despite their high prevalence, the molecular mechanisms underlying fibroid pathogenesis remain incompletely understood.

Our group was the first to report a marked dysregulation of the tryptophan (Trp) catabolic pathway in uterine fibroids [3,4], a feature previously described in multiple malignancies [5–10]. We proposed that this metabolic reprogramming may contribute to immune evasion in fibroids through mechanisms similar to those observed in cancer.

Trp, an essential amino acid, is primarily metabolized by tryptophan 2,3-dioxygenase (TDO2) and indoleamine 2,3-dioxygenase 1 (IDO1) to generate kynurenine (KYN), a biologically active metabolite that activates the aryl hydrocarbon receptor (AhR). Upon ligand binding, AhR translocates to the nucleus and binds to aryl response elements, thereby regulating the transcription of downstream target genes. Our previous studies demonstrated that TDO2, rather than IDO1, is consistently overexpressed in fibroids, particularly in MED12-mutated tumors and tumors from Black patients, and that fibroids exhibit significantly elevated KYN levels compared with matched myometrium, indicating increased enzymatic activity [3]. We further showed dysregulation of additional components of the Trp pathway, including overexpression of Trp transporters (SLC7A5, SLC7A8), increased expression of the AhR target gene CYP1B1, and reduced expression of the KYN-degrading enzyme KYNU [4]. These alterations reflect a metabolic shift favoring KYN production over NAD synthesis, consistent with reduced NAD levels observed in fibroids.

Functional studies supported the dominant role of TDO2 in fibroid biology. Inhibition of TDO2, but not IDO1, suppressed COL1A1 and COL3A1 expression in fibroid spheroids [3]. More recently, we demonstrated that pharmacologic inhibition of TDO2 in mice bearing fibroid xenografts resulted in approximately 30% tumor regression, reduced cellular proliferation, and decreased expression of multiple AhR-regulated genes implicated in fibroid pathogenesis, including TGF-β3, FN1, CDK2, E2F1, IL-8, and SPARC [11].

In the present study, we performed next-generation sequencing (NGS) of large and small RNAs in fibroid xenografts following 2 months of TDO2 inhibitor (680C91) treatment, followed by validation of selected targets using qRT-PCR, protein analyses, and *in vitro* fibroid explant models, to further define the transcriptomic landscape underlying TDO2-mediated fibroid regression.

## Materials and methods

### Fibroid specimen collection

Intramural uterine fibroids (2–5 cm in diameter) were collected from hysterectomy specimens obtained at Harbor UCLA Medical Center from patients undergoing surgery for symptomatic fibroids (*n* = 76–116). The study was approved by the Institutional Review Board of The Lundquist Institute (IRB #18CR-31752-01R), and written informed consent was obtained from all participants. Samples were obtained exclusively from premenopausal patients who had not used hormonal medications for at least 3 months prior to surgery. Donor age ranged from 30 to 50 years (mean ± SD: 43 ± 4.6 years). A portion of each tumor was allocated for *in vivo* studies, and remaining tissue was snap-frozen and stored in liquid nitrogen as previously described [12,13].

### MED12 mutation analysis

Genomic DNA was extracted from fibroid tissues and paired myometrium (100 mg per sample) using the MagaZorb DNA Mini-Prep Kit (Promega, Madison, WI, U.S.A.) according to the manufacturer’s instructions. PCR amplification of MED12 exon 2 was performed using the following primers (5′–3′): forward, GCCCTTTCACCTTGTTCCTT, and reverse, TGTCCCTATAAGTCTTCCCAACC. PCR products were subjected to Sanger sequencing (Laragen Inc., Culver City, CA, U.S.A.) using BigDye Terminator v3.1 sequencing chemistry. Sequence chromatograms were analyzed using ChromasPro software (version 2.1.8) and aligned to the reference MED12 genomic (NG_012808) and transcript (NM_005120) sequences to identify mutations.

### Fibroid xenograft model

All animal procedures were conducted under an approved protocol (32133-01) by the Institutional Animal Care and Use Committee at the Lundquist Institute at Harbor-UCLA Medical Center. Female ovariectomized SCID/Beige mice (9–12 weeks old; Charles River Laboratories, Hollister, CA, U.S.A.) were supplemented with slow-release hormone pellets (Innovative Research of America, Sarasota, FL, U.S.A.) containing estradiol (0.075 mg, 60-day release) and progesterone (75 mg, 60-day release), as previously described [11]. Fresh human fibroid tissue (∼0.5 g per specimen) was aseptically sectioned into 5–10 small fragments and subcutaneously implanted into the flanks of recipient mice. Fibroid tissues obtained from 12 patients were divided into fragments and implanted into two recipient mice per patient. One mouse per pair received vehicle treatment, while the corresponding mouse received 680C91, allowing for within-patient comparisons. A total of 24 mice were used. Following a 3-day post-surgical recovery period, mice were administered daily intraperitoneal injections of either the TDO2 inhibitor 680C91 (10 mg/kg) or vehicle. 680C91 (Cayman Chemical, Ann Arbor, MI, U.S.A.) was first dissolved in DMSO (0.05 mg/μl) and subsequently diluted in saline to achieve a final DMSO:saline ratio of 11:9, with a total injection volume of 100 μl per mouse. After 8 weeks of treatment, animals were euthanized, and blood samples were collected via cardiac puncture. Xenograft tumors were excised, carefully separated from adjacent tissues, weighed, and snap-frozen for downstream analyses.

### RNA sequencing and bioinformatic analysis

Total RNA was isolated from fibroid xenografts. Fibroid fragments from each patient were divided and implanted into separate mice, which were subsequently treated with either 680C91 or vehicle, as previously described [11], using TRIzol reagent (Thermo Fisher Scientific, Waltham, MA, U.S.A.). RNA concentration was measured with a NanoDrop 2000c spectrophotometer (Thermo Scientific, Wilmington, DE, U.S.A.), and RNA integrity was assessed using an Agilent 2100 Bioanalyzer (Agilent Technologies, Santa Clara, CA, U.S.A.) [14,15]. RNA samples from three independent patient-derived xenografts (*n* = 3 biological replicates per group), each with RNA integrity number ≥9, were used for library preparation. For large RNA sequencing, 1 μg of total RNA per sample was used to generate strand-specific cDNA libraries, and 500 ng per sample was used to prepare small RNA libraries using the TruSeq protocol (Illumina, San Diego, CA, U.S.A.). Barcoded libraries were pooled and sequenced on an Illumina MiSeq platform using a 35-bp single-end format, producing ∼10 million reads per library with alignment efficiencies of 80–90% [14,15]. Sequencing and primary processing were performed at the UCLA Technology Center for Genomics & Bioinformatics. Raw sequencing reads were quality-checked and aligned to the human reference genome (e.g., hg38) using HISAT2. Gene-level read counts were generated using featureCounts (subread). Differential expression analysis was performed using DESeq2, which applies a model based on the negative binomial distribution. Read counts were normalized using DESeq2’s internal size factor normalization to account for sequencing depth and library composition. Statistical significance was defined as an adjusted *P*-value (false discovery rate, FDR) <0.05 and fold change ≥1.5. Downstream analyses included hierarchical clustering, TreeView visualization, volcano plots, principal component analysis (PCA), and functional enrichment using Flaski [16] and RNAenrich [17]. Protein–protein interaction (PPI) networks were constructed using the STRING database [18]. All datasets met predefined quality control criteria for differential expression analysis. The RNA sequencing data have been deposited in the Gene Expression Omnibus (GEO) database under accession number GSE319228.

### Fibroid explant culture

Fibroid explants were aseptically dissected from the same patient and plated as equal-weight pieces in six-well plates containing complete medium. Explants were treated for 48 h with vehicle or 680C91 (50 μM).

### Primary cell culture and siRNA transfection

Primary myometrial smooth muscle cells (MSMC) and fibroid smooth muscle cells (LSMC) were isolated from freshly obtained matched myometrial and fibroid tissues, as previously described [19]. Cells were maintained in Dulbecco’s modified Eagle medium supplemented with 10% fetal bovine serum and cultured under standard conditions, with medium replacement every 2–3 days until reaching confluence. Experiments were conducted using early passage cells (passages 1–3). All cell culture studies were independently repeated at least three times using cells derived from different patient samples. All reagents and consumables for tissue processing and cell culture were obtained from Sigma–Aldrich (St. Louis, MO, U.S.A.), Invitrogen (Carlsbad, CA, U.S.A.), and Fisher Scientific (Atlanta, GA, U.S.A.). For gene silencing experiments, primary LSMCs were transfected with 50 nM of either a non-targeting control siRNA (siNC) or siRNA targeting TDO2 (siTDO2; 5′-CUAUCACUACCUGCGAUCAACUGUG-3′) using PureFection transfection reagent (System Biosciences, Mountain View, CA, U.S.A.), according to the manufacturer’s protocol.

### Generation of lentiviral TDO2 overexpression cells

A lentiviral expression vector encoding full-length human TDO2 (pReceiver-Lv233; Cat# EX-C0452-Lv233) and the corresponding empty control vector (Cat# EX-NEG-Lv233) were obtained from GeneCopoeia (Rockville, MD, U.S.A.). Lentiviral particles were produced by co-transfecting human embryonic kidney 293T cells with the transfer plasmid along with the packaging plasmids psPAX2 (Addgene #12260) and pMD2.G (Addgene #12259), using PureFection transfection reagent (System Biosciences, Mountain View, CA, U.S.A.) according to the manufacturer’s instructions. Viral supernatants were collected daily for up to 72 h post-transfection, clarified by centrifugation, and used to transduce primary MSMC in the presence of polybrene (8 μg/ml). Forty-eight hours after transduction, cells were subjected to selection with puromycin (0.5 μg/ml) to establish stable populations. Cells were maintained under selection and harvested 7 days after the initial transduction for downstream analyses.

### Quantitative RT-PCR

Total RNA was extracted from fibroid xenografts, explants, and from paired fibroid and matched myometrium tissues using TRIzol reagent (Thermo Fisher Scientific). RNA concentration and purity were measured using a NanoDrop 2000c spectrophotometer, and integrity was assessed using an Agilent 2100 Bioanalyzer, as previously described [20]. For cDNA synthesis, 1 μg of total RNA was reverse-transcribed using random hexamer primers (Applied Biosystems, Carlsbad, CA, U.S.A.). Quantitative PCR was performed using SYBR Gene Expression Master Mix (Applied Biosystems) on an Applied Biosystems 7500 Fast Real-Time PCR System. Gene expression was normalized to RNU6-2 for small RNA assays and FBXW2 for large RNA assays. Reactions were run in technical triplicate, and relative expression was calculated using the 2^−ΔΔCt^ method. Primer sequences are provided in Supplementary Table S1.

### Immunoblotting

Protein extracts from fibroid explants and paired fibroid and matched myometrium tissues were analyzed by immunoblotting as previously described [19]. Primary antibodies against COL11A1, MMP11, MMP14, VDR, α-smooth muscle actin (αSMA), vimentin (VIM), and phosphorylated AKT (Ser473) were obtained from Proteintech Group, Inc. (Chicago, IL, U.S.A.), and CBX4 antibody was obtained from Santa Cruz Biotechnology (Dallas, TX, U.S.A.). Band intensities were quantified using ImageJ and normalized to ACTB (Proteintech Group, Inc.). Data are presented as mean ± SEM and expressed relative to the control group (set to 1).

### Statistical analysis

All quantitative results are presented as mean ± SEM. Statistical analyses were performed using GraphPad Prism (GraphPad Software, San Diego, CA, U.S.A.). Data distributions were assessed using the Kolmogorov–Smirnov test. Because the datasets did not meet normality assumptions, nonparametric tests were used. Comparisons between two groups were performed using the Wilcoxon matched-pairs signed-rank test. A two-sided *P* <0.05 was considered statistically significant.

## Results

### High-throughput sequencing of coding and non-coding RNAs in fibroid xenografts treated with vehicle or a TDO2 inhibitor

**Figure 1:**
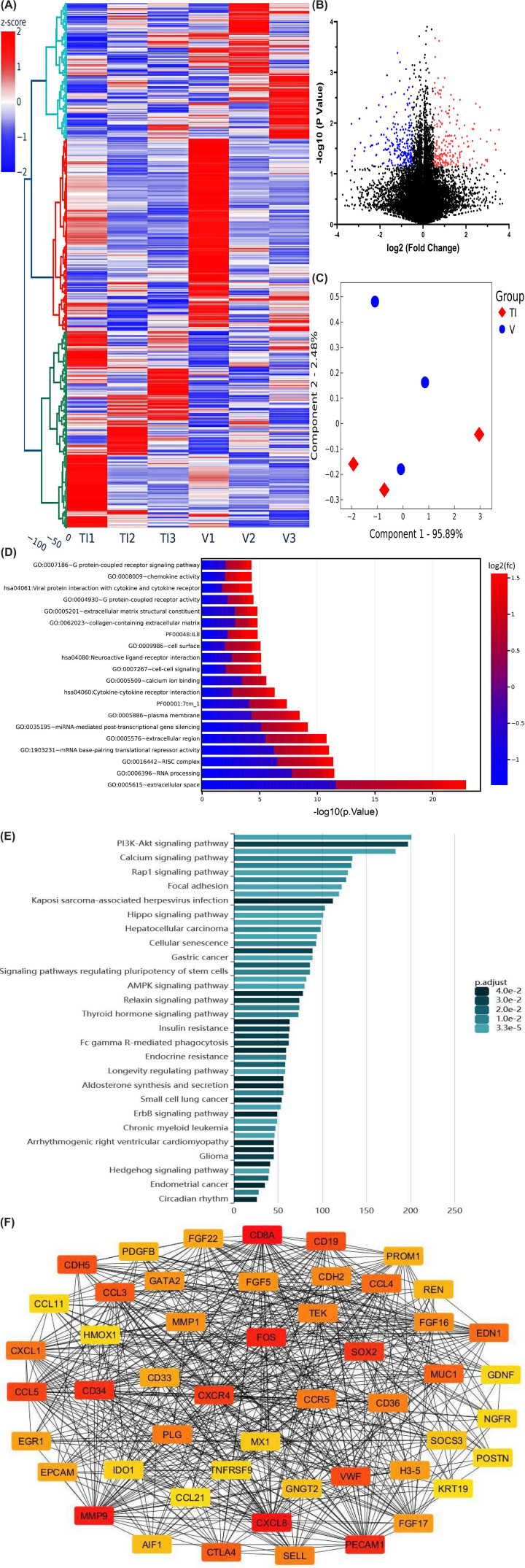
Transcriptomic profiling of fibroid xenografts following TDO2 inhibitor treatment (**A**) Hierarchically clustered heatmap showing differentially expressed transcripts (fold change ≥1.5, *P* <0.05) in fibroid xenografts after 8 weeks of treatment with vehicle (V) or the TDO2 inhibitor 680C91 (TI; *n* = 3). Gene expression levels are displayed as *z*-scores. (**B**) Volcano plot illustrating significantly up-regulated (*n* = 245; red) and down-regulated (*n* = 252; blue) transcripts using an FDR-adjusted *P* <0.05. (**C**) PCA of RNA-seq data from three independent patient-derived xenografts treated with vehicle (blue) or TDO2 inhibitor (red). Each point represents an individual sample (*n* = 3 per group). (**D,E**) GO and KEGG pathway enrichment analyses of differentially expressed coding RNAs (**D**) and lncRNAs (**E**) in the TDO2 inhibitor-treated group. (**F**) PPI network of the top 50 hub genes, constructed using the STRING database and Cytoscape software (version 3.10.2). Node colors indicate interaction degree (red to yellow, highest to lowest).

Our previous studies demonstrated that treatment with the TDO2 inhibitor 680C91 significantly reduced fibroid xenograft weight after two months and suppressed the expression of CYP1B1, TGF-β3, FN1, CDK2, E2F1, IL-8, SPARC, and Ki67 compared with vehicle-treated controls [11]. Building on these findings, we performed high-throughput NGS on RNA isolated from three independent patient-derived xenografts treated with 680C91 or vehicle. After normalization of 28 573 RNA transcripts, hierarchical clustering and TreeView analyses identified 5433 differentially expressed transcripts, including 2040 up-regulated and 3393 down-regulated transcripts (≥1.5-fold) in the TDO2 inhibitor-treated group (TI) compared with matched vehicle controls (V) (Figure 1A). Among these, 3449 were coding RNAs (1302 up-regulated and 2147 down-regulated) and 826 were long non-coding RNAs (lncRNAs) (290 up-regulated and 536 down-regulated). Volcano plot analysis further identified 245 significantly up-regulated and 252 significantly down-regulated transcripts (fold change ≥1.5, *P* <0.05) (Figure 1B). PCA followed by k-means clustering demonstrated clear separation between TI and V groups, indicating high data reliability (Figure 1C). Gene Ontology (GO) and KEGG (Kyoto Encyclopedia of Genes and Genomes) pathway enrichment analyses of coding RNAs (Figure 1D) and lncRNAs (Figure 1E) revealed predominant involvement in the extracellular space, RNA processing, PI3K/AKT signaling, and calcium signaling pathways. PPI network analysis using the STRING database and Cytoscape, focusing on the top 50 hub genes identified by the CytoHubba plugin, highlighted key molecular interactions associated with TDO2 inhibition (Figure 1F). Several proteins identified in the PPI network, including IDO1, PDGFB, MUC1, CXCL8, and FOS, are well established in fibroid pathogenesis, whereas others such as FGF17, FGF22, SOX2, REN, PROM1, and GATA2 represent novel candidates warranting further investigation.

### Small non-coding RNA profiling in TDO2 inhibitor-treated fibroid xenografts

**Figure 2:**
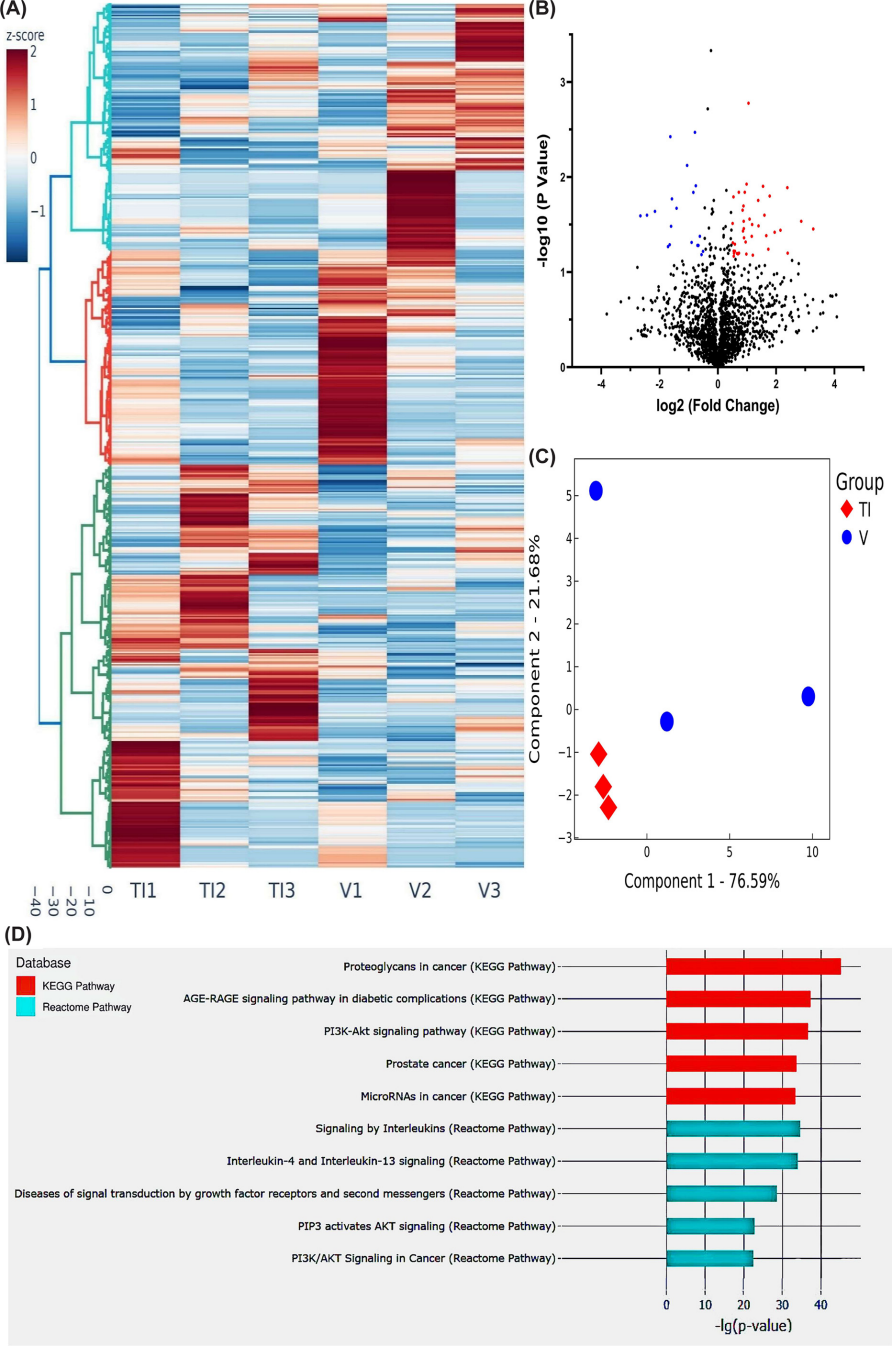
SncRNA profiling of fibroid xenografts following TDO2 inhibitor treatment (**A**) Hierarchically clustered heatmap showing differentially expressed sncRNAs (fold change ≥1.5, *P* <0.05) in fibroid xenografts treated with vehicle (V) or the TDO2 inhibitor 680C91 (TI; *n* = 3). Expression levels are displayed as *z*-scores. (**B**) Volcano plot illustrating significantly up-regulated (red; *n* = 40) and down-regulated (blue; *n* = 20) sncRNAs using an FDR-adjusted *P* <0.05.(**C**) PCA of sncRNA-seq data from vehicle-treated (blue) and TDO2 inhibitor-treated (red) fibroid xenografts (*n* = 3 per group). Each point represents an individual sample. (**D**) GO and KEGG pathway enrichment analyses of differentially expressed miRNAs in vehicle versus TDO2 inhibitor-treated groups.

Using NGS-based small non-coding RNA (sncRNA) sequencing, we next characterized sncRNA expression changes in the TI group compared with matched vehicle controls. After normalization and analysis using the AASRA database, a total of 1922 sncRNAs were identified. Hierarchical clustering revealed 776 significantly altered sncRNAs, including 362 up-regulated and 414 down-regulated transcripts (≥1.5-fold) in the TI group (Figure 2A). These included 11 snRNAs, 108 snoRNAs, 337 miRNAs, 156 piRNAs, 9 tRNAs, and 34 rRNAs. Volcano plot analysis identified 40 significantly up-regulated and 20 significantly down-regulated sncRNAs (*P* <0.05) (Figure 2B). PCA and k-means clustering demonstrated distinct sncRNA expression patterns between TI and V groups, confirming data robustness (Figure 2C). Pathway enrichment analysis using RNAenrich, based on KEGG and Reactome databases, showed that the 135 mapped miRNAs were primarily associated with PI3K/AKT signaling, proteoglycans in cancer, and interleukin signaling pathways (Figure 2D).

### Validation of TDO2 inhibitor-associated coding and non-coding RNAs in fibroid explants and xenografts

**Figure 3:**
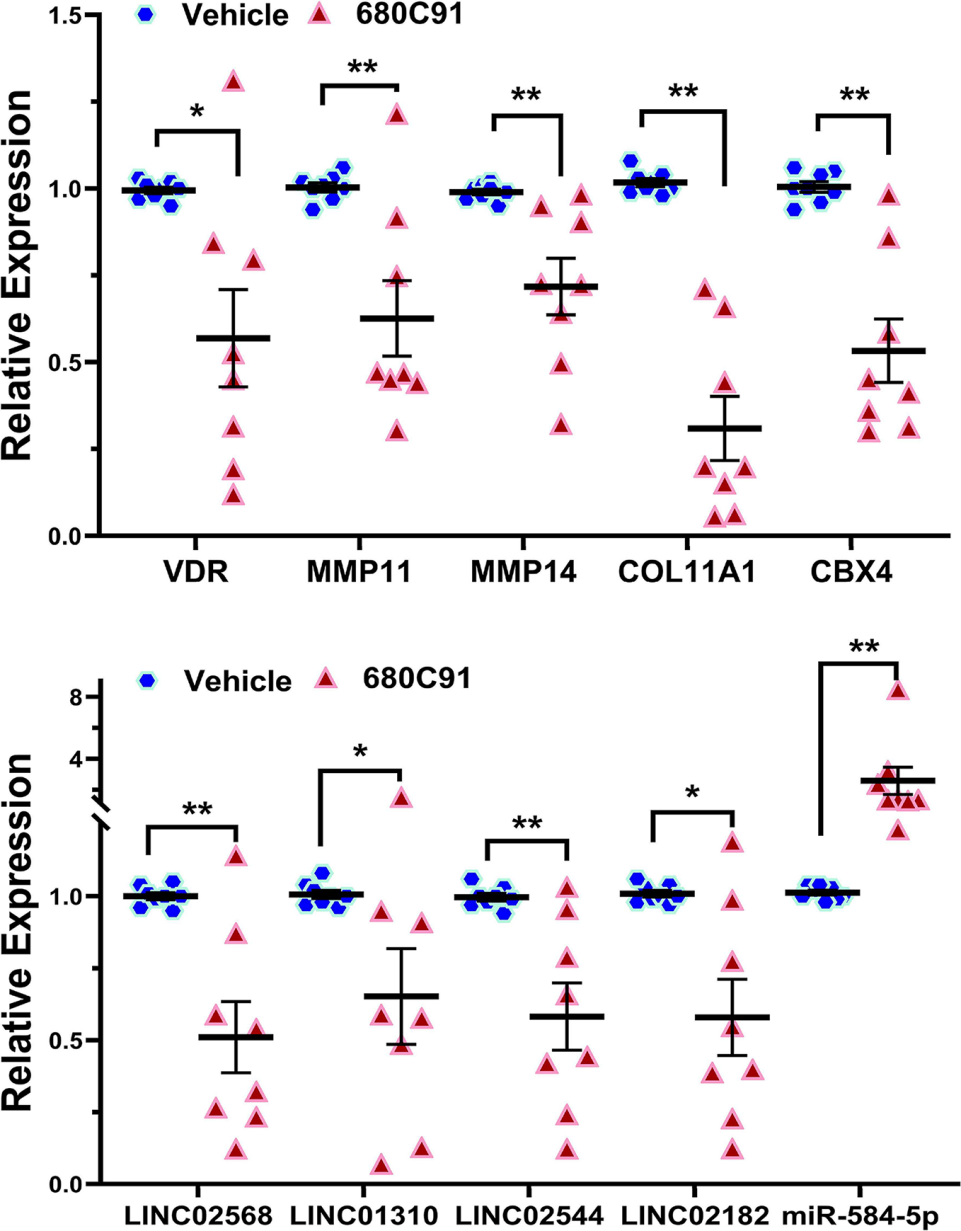
Effects of TDO2 inhibition on the expression of coding genes and non-coding RNAs in fibroid xenografts. Validation of TDO2 inhibitor-regulated transcripts in fibroid xenografts. Relative mRNA expression levels of VDR, MMP11, MMP14, COL11A1, CBX4, LINC02568, LINC01310, LINC02544, LINC02182, and miR-584-5p in fibroid xenografts implanted subcutaneously in ovariectomized CB-17 SCID/Beige mice (*n* = 8) following 8 weeks of treatment with vehicle or the TDO2 inhibitor 680C91 (10 mg/kg). Data are presented as mean ± SEM, with *P*-values indicated by connecting lines. *P <0.05; **P <0.01.

Given the exploratory nature of this analysis and the limited sample size, we further focused our interpretation on genes consistently altered across samples and supported by independent validation. Ten novel coding and non-coding RNAs were selected and evaluated for their expression by qRT-PCR in fibroid xenografts (*n* = 8) from mice treated with 680C91 or vehicle. Two months of 680C91 treatment resulted in significant down-regulation of VDR, MMP11, MMP14, COL11A1, CBX4, LINC02568, LINC01310, LINC02544, and LINC02182, along with significant up-regulation of miR-584-5p (Figure 3).

**Figure 4:**
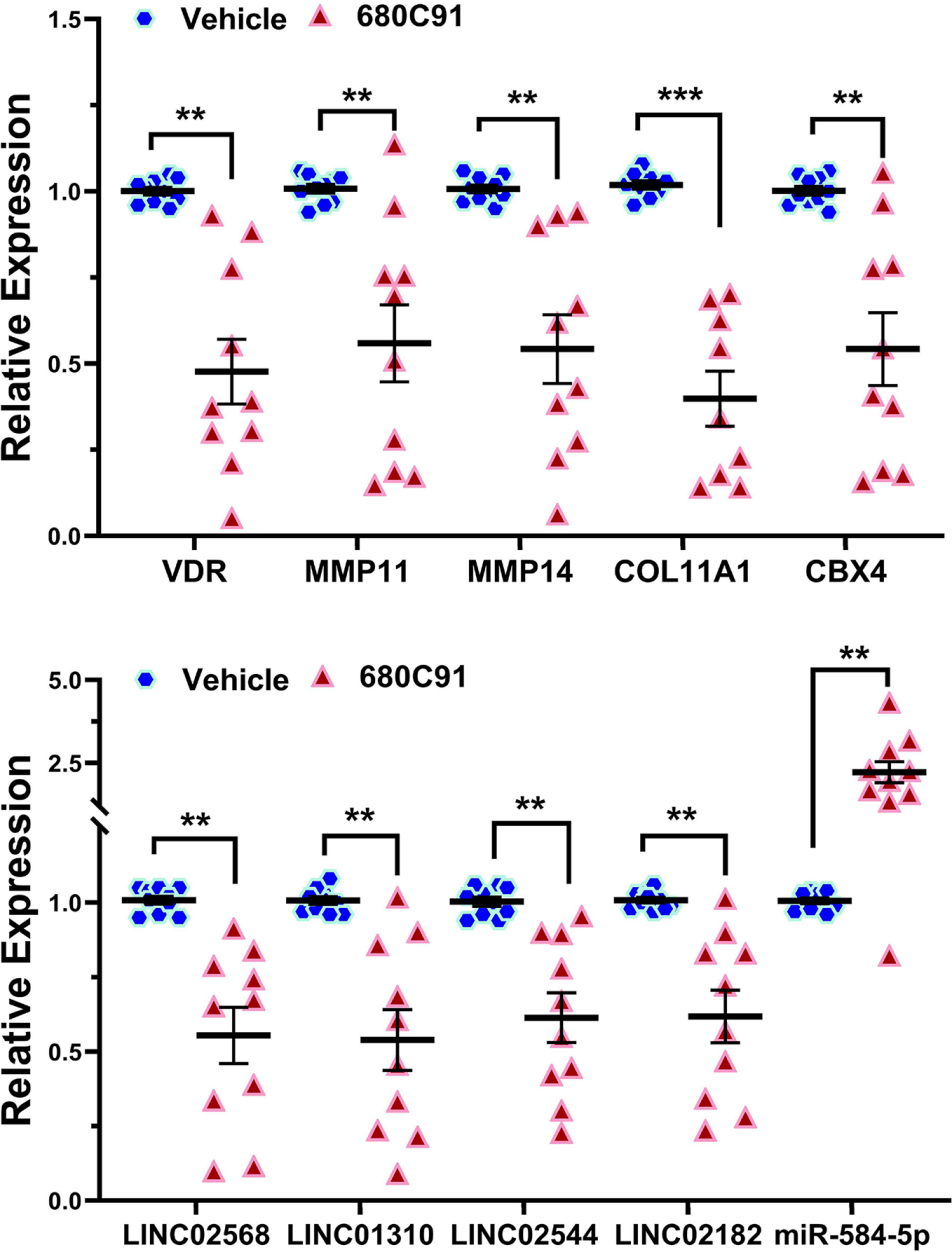
TDO2 inhibition modulates expression of coding and non-coding transcripts in fibroid explants. Validation of TDO2 inhibitor-regulated transcripts in fibroid explants. Fibroid explants (*n* = 10) were treated with vehicle or the TDO2 inhibitor 680C91 (50 μM) for 48 h, and mRNA expression levels were quantified by real-time quantitative PCR. Data are presented as mean ± SEM, with *P*-values indicated by connecting lines. **P <0.01; ***P <0.001.

**Figure 5:**
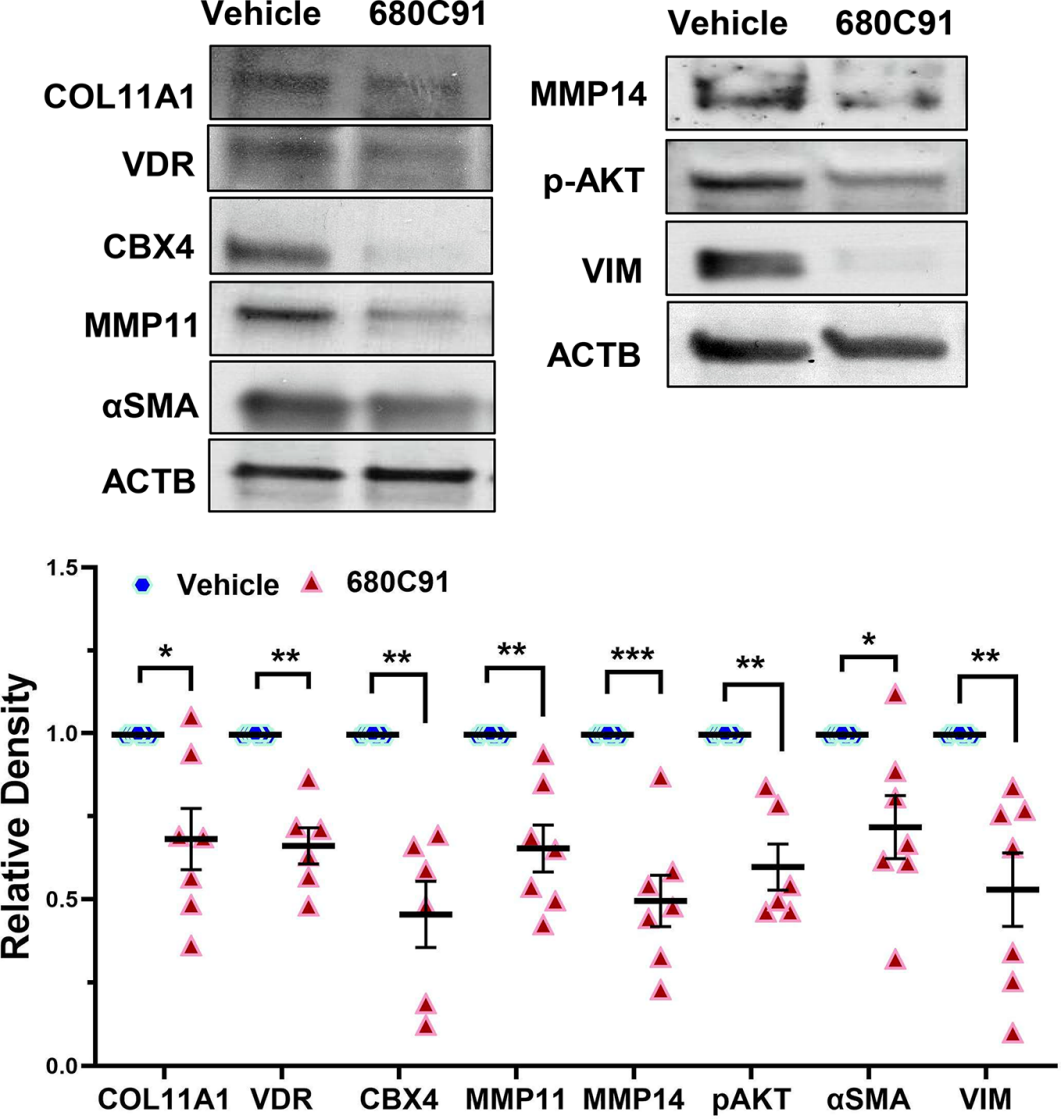
In vivo validation of TDO2 inhibitor-regulated protein expression in fibroid xenografts. Protein expression changes in fibroid xenografts following TDO2 inhibitor treatment. Representative western blot analyses of VDR, MMP11, MMP14, COL11A1, CBX4, phosphorylated AKT (Ser473), αSMA, and VIM, with accompanying bar graphs showing relative band densitometry in fibroid xenografts (*n* = 7). Data are presented as mean ± SEM from independent experiments, with *P*-values indicated by connecting lines. *P <0.05; **P <0.01; ***P <0.001.

**Figure 6:**
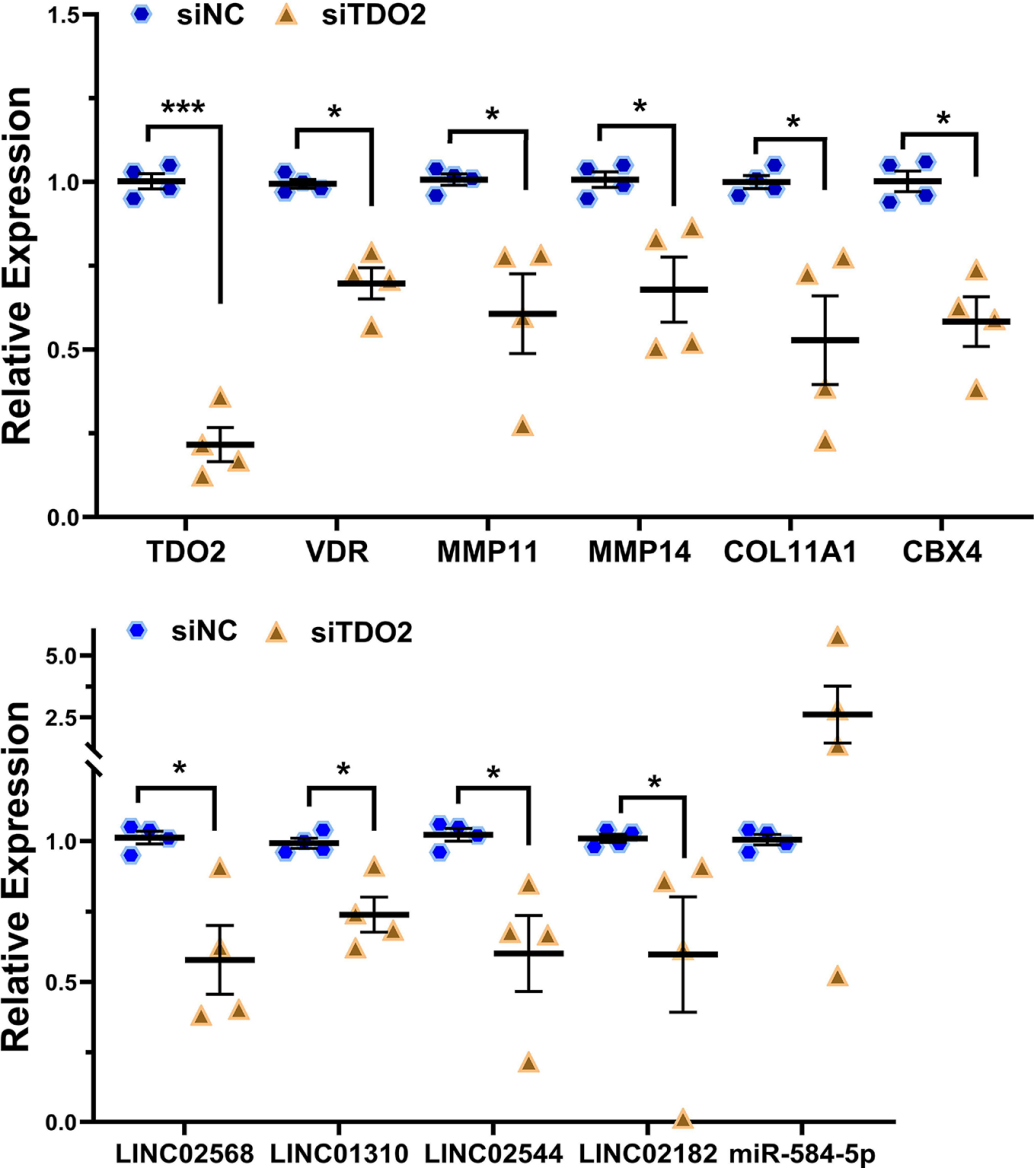
Functional validation of TDO2-regulated coding and non-coding transcripts in primary LSMCs. Effects of TDO2 silencing in primary LSMC. Cells were transfected with siTDO2 or control siRNA (siNC) for 96 h, followed by assessment of gene expression by qRT-PCR. Expression levels of TDO2, VDR, MMP11, MMP14, COL11A1, CBX4, LINC02568, LINC01310, LINC02544, LINC02182, and miR-584-5p are shown. Data represent mean ± SEM from four independent experiments (*n* = 4). Statistical significance is indicated as *P <0.05 and ***P <0.01.

**Figure 7:**
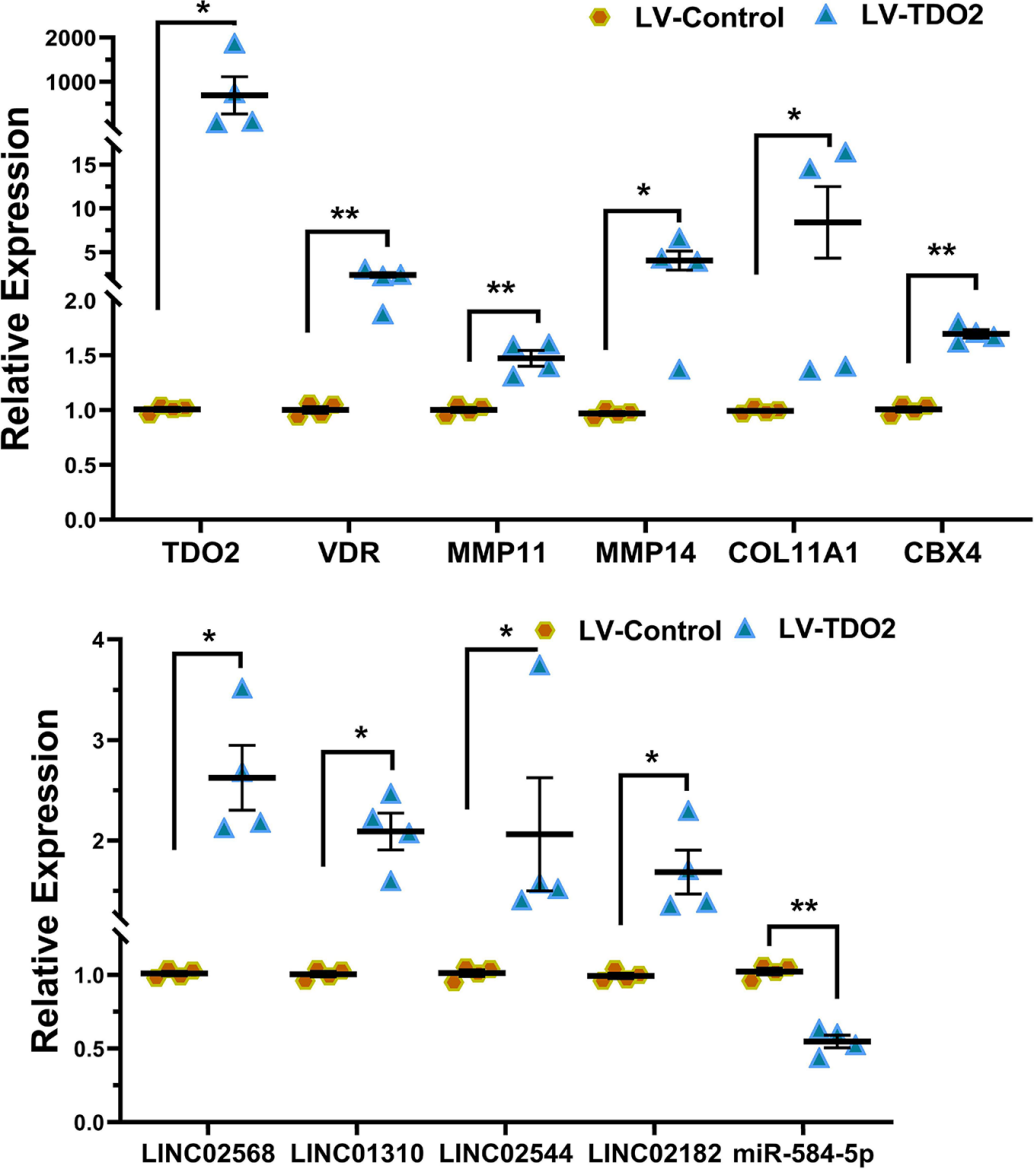
Gain-of-function analysis of TDO2-regulated coding and non-coding transcripts in primary MSMCs. Gene expression changes following TDO2 overexpression in primary MSMC. Cells were transduced with lentiviral vectors encoding TDO2 (LV-TDO2) or empty control (LV-Control), and transcript levels were quantified by qRT-PCR. Relative expression of TDO2, VDR, MMP11, MMP14, COL11A1, CBX4, LINC02568, LINC01310, LINC02544, LINC02182, and miR-584-5p is presented. Values are expressed as mean ± SEM (*n* = 4). Significance levels are denoted as *P <0.05 and **P <0.01.

Consistent results were obtained in *in vitro* fibroid explant studies (*n* = 10), where treatment with 680C91 (50 μM) for 48 h produced the same expression pattern observed in xenografts. Specifically, mRNA levels of VDR, MMP11, MMP14, COL11A1, CBX4, LINC02568, LINC01310, LINC02544, and LINC02182 were significantly reduced, while miR-584-5p expression was increased in TDO2 inhibitor-treated explants compared with vehicle controls (Figure 4), confirming the RNA-seq results. Protein analysis further supported these findings. Treatment of fibroid explants with 680C91 significantly reduced protein levels of COL11A1, VDR, CBX4, MMP11, MMP14, phosphorylated AKT, αSMA, and VIM compared with vehicle-treated explants (Figure 5). To further assess the specificity of TDO2 inhibition, we performed genetic modulation of TDO2 expression. siRNA-mediated knockdown of TDO2 significantly decreased mRNA levels of VDR, MMP11, MMP14, COL11A1, CBX4, LINC02568, LINC01310, LINC02544, and LINC02182, while miR-584-5p expression showed an increasing trend compared with siRNA control (Figure 6). These changes were consistent with those observed following pharmacological inhibition with 680C91. In contrast, lentiviral overexpression of TDO2 produced opposing effects on these transcripts (Figure 7). Collectively, these findings support the conclusion that the observed molecular changes are specifically mediated by TDO2 activity.

### Expression of validated genes in fibroid and matched myometrium

**Figure 8:**
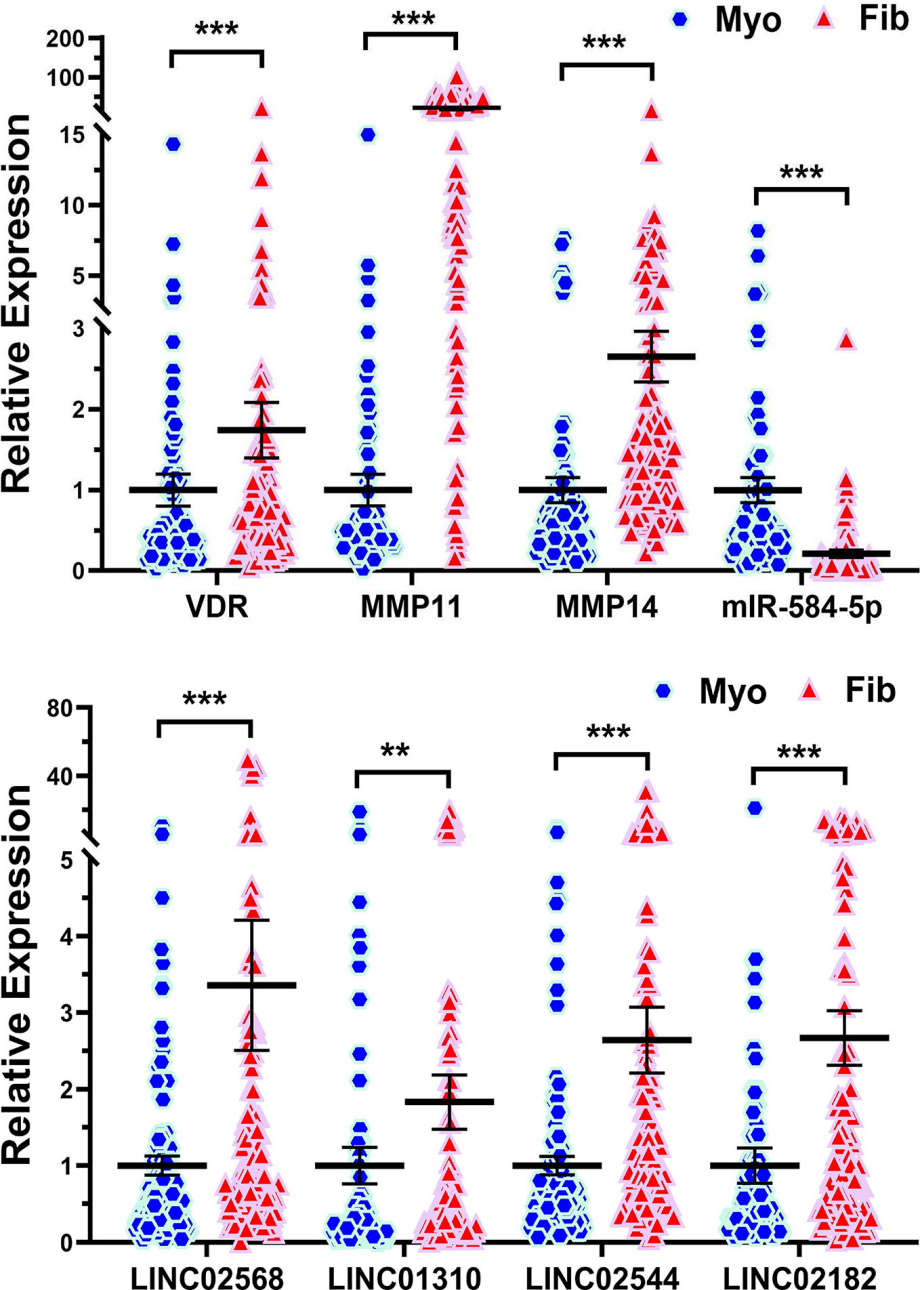
Validation of TDO2-associated coding and non-coding transcript dysregulation in human fibroid tissues. Differential expression of validated transcripts in paired myometrium and fibroid tissues. Relative expression levels of VDR (*n* = 85), MMP11 (*n* = 86), MMP14 (*n* = 86), LINC02568 (*n* = 115), LINC01310 (*n* = 96), LINC02544 (*n* = 116), LINC02182 (*n* = 94), and miR-584-5p (*n* = 76) were measured by qRT-PCR in paired myometrium and fibroid samples. Data are presented as mean ± SEM, with statistical significance indicated by connecting lines (*P <0.05; **P <0.01; ***P <0.001).

**Figure 9:**
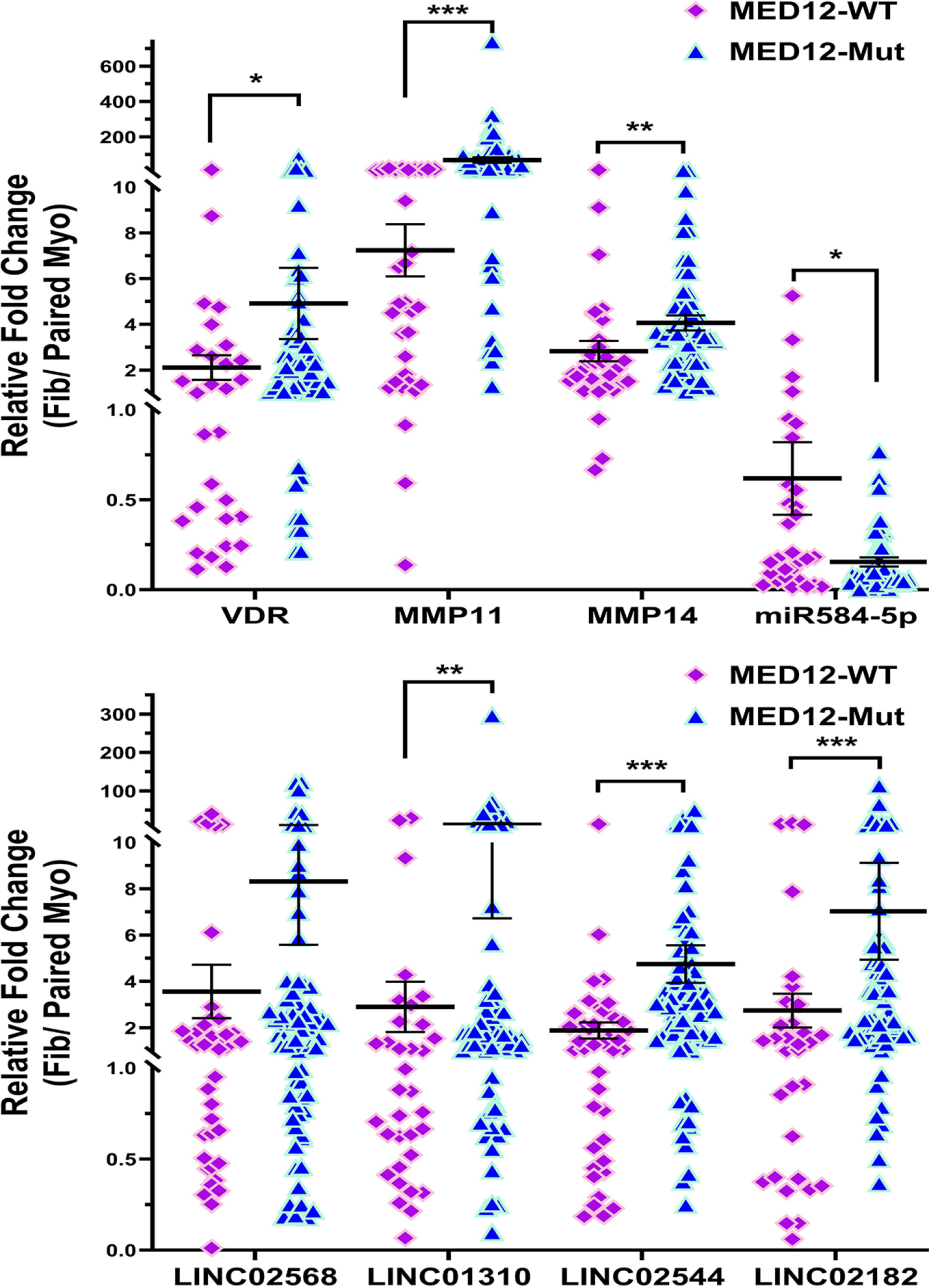
MED12 mutation status influences expression of TDO2-associated coding and non-coding transcripts in fibroids. Differential expression of validated transcripts stratified by MED12 mutation status. Relative expression of VDR, MMP11, MMP14, LINC02568, LINC01310, LINC02544, LINC02182, and miR-584-5p, expressed as fold change (fibroid/paired myometrium), was measured by qRT-PCR in MED12-mutated (*n* = 46–75) and non-mutated (*n* = 29–41) specimens. Data are presented as mean ± SEM, with statistical significance indicated by connecting lines (*P <0.05; **P <0.01; ***P <0.001).

**Figure 10:**
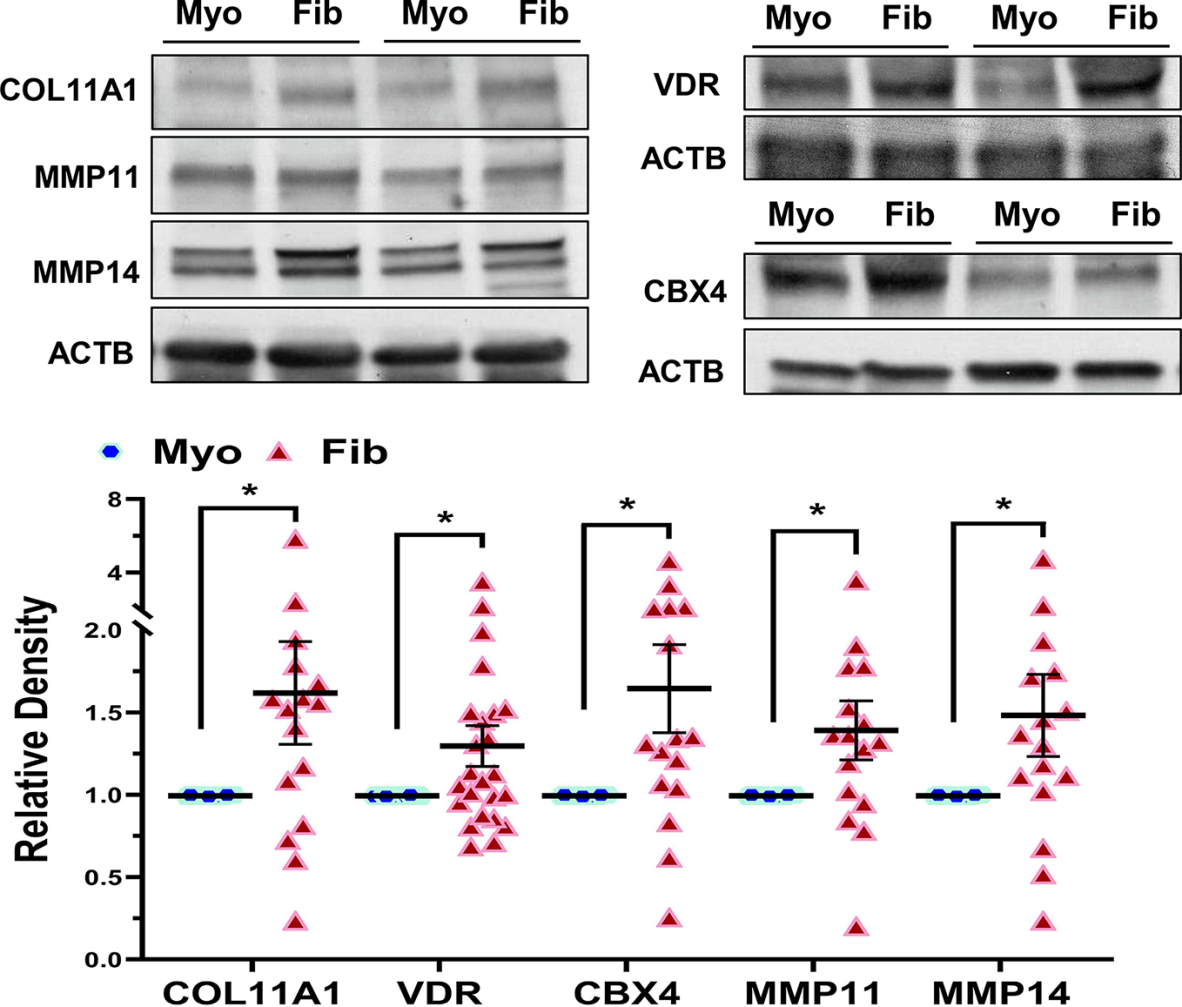
Validation of TDO2-associated protein dysregulation in human fibroid tissues. Protein expression of validated targets in paired myometrium and fibroid tissues. Representative western blot analyses of VDR, COL11A1, MMP11, MMP14, and CBX4, with accompanying bar graphs showing relative band densitometry in paired myometrium and fibroid samples (*n* = 16–24). Data are presented as mean ± SEM, with statistical significance indicated by connecting lines (*P <0.05; **P <0.01).

To assess whether validated targets were differentially expressed in human fibroid tissue, we measured mRNA levels in a large cohort of paired fibroid and matched myometrium samples (*n* = 76–116). This analysis showed significantly higher expression of VDR, MMP11, MMP14, LINC02568, LINC01310, LINC02544, and LINC02182 in fibroids, whereas miR-584-5p levels were significantly lower compared with matched myometrium (Figure 8). Stratification by MED12 mutation status revealed that fold-change expression (fibroid/paired myometrium) of VDR, MMP11, MMP14, LINC01310, LINC02544, and LINC02182 was significantly higher, while miR-584-5p expression was significantly lower, in MED12-mutated fibroids compared with non-mutated tumors (Figure 9). As the expression of COL11A1 and CBX4 in fibroids has been previously reported [20,21], these data are not shown in Figures 8 and 9. Protein expression analysis in paired fibroid and myometrium tissues (*n* = 16–24) demonstrated significant up-regulation of VDR, MMP11, MMP14, COL11A1, and CBX4 in fibroids (Figure 10). Collectively, these results demonstrate that all validated transcripts exhibit directional expression changes opposite to those observed following TDO2 inhibition, further supporting the role of TDO2 in fibroid pathogenesis.

## Discussion

The results of the present study demonstrate that long-term inhibition of TDO2 induces widespread transcriptomic reprogramming in uterine fibroids, affecting both coding and non-coding RNAs. Large RNA sequencing revealed enrichment of pathways related to the extracellular space, RNA processing, PI3K/AKT signaling, and calcium signaling, whereas small RNA sequencing identified enrichment of pathways involving PI3K/AKT signaling, proteoglycans in cancer, and interleukin signaling. Selected differentially expressed genes identified by RNA sequencing were validated in fibroid xenografts treated with 680C91 and in *in vitro* fibroid explant models, confirming the robustness of the sequencing data. Treatment with 680C91 significantly suppressed the expression of VDR, MMP11, MMP14, COL11A1, CBX4, LINC02568, LINC01310, LINC02544, and LINC02182, while increasing miR-584-5p expression at both mRNA and protein levels. Although 680C91 has reported limitations related to solubility and potential off-target effects, our study addressed this concern using additional genetic approaches. Specifically, siRNA-mediated knockdown of TDO2 recapitulated the effects of pharmacological inhibition, while TDO2 overexpression produced opposite changes in target gene expression. These findings support the specificity of TDO2 as a key regulator of the observed transcriptomic alterations. Importantly, all validated transcripts exhibited directional expression changes opposite to those observed in fibroid tissues compared with matched myometrium, with more pronounced differences in MED12-mutated tumors, highlighting their relevance to fibroid pathogenesis.

A defining feature of uterine fibroids is excessive extracellular matrix (ECM) accumulation [22]. Our data indicate that TDO2 inhibition preferentially targets genes involved in ECM composition and remodeling, including collagens, proteoglycans, and matrix metalloproteinases. We previously reported that 680C91 suppresses COL1A1, COL3A1 [3], and FN1 [11], a major ECM glycoprotein that interacts with cell-surface proteoglycans. In the present study, we validated the expression of MMP11 and MMP14, both of which are up-regulated in fibroids [23,24]. MMP14 is also elevated in leiomyosarcoma [25]. In multiple cancers, MMP11 and MMP14 promote tumor invasion, migration, angiogenesis, and metastasis through ECM degradation and activation of additional MMPs like MMP2 and MMP9 [26–30]. The suppression of these enzymes by TDO2 inhibition supports a mechanism by which ECM remodeling and fibrotic progression are attenuated in fibroids. Importantly, the transcriptomic changes observed in the present study are consistent with previously reported functional outcomes following TDO2 inhibition, including reduced tumor growth, decreased collagen deposition, and diminished cell proliferation in fibroid xenografts [11]. These findings support the biological relevance of the identified pathways, particularly those related to extracellular matrix remodeling and cell cycle regulation. Together, these data suggest that the molecular alterations identified here contribute to the observed anti-fibrotic and anti-proliferative effects of TDO2 inhibition.

Consistent with this interpretation, 680C91 significantly reduced the expression of αSMA and VIM, both of which are overexpressed in fibroids [31–33]. αSMA is a marker of activated myofibroblasts, the primary ECM-producing cells in fibroids [34]. Reduced αSMA expression suggests a shift away from a profibrotic, matrix-producing phenotype toward a more quiescent state [35]. VIM, a key cytoskeletal protein involved in mechanosensing and fibroblast function, is essential for ECM remodeling and wound healing [36,37]. Reduced VIM expression has been associated with impaired fibroblast proliferation and decreased collagen deposition [38,39]. Together, these findings support the conclusion that TDO2 inhibition disrupts fibrotic signaling at multiple levels.

RNA sequencing also identified the PI3K/AKT pathway as a major target of TDO2 inhibition, which was confirmed by reduced AKT phosphorylation in fibroid explants. The PI3K/AKT pathway is a central regulator of cell proliferation, survival, angiogenesis, and metabolism [40,41], and its activation has been well documented in fibroids [42,43]. AKT and ERK signaling are also critical for fibroblast activation and migration [38,39]. Thus, inhibition of AKT phosphorylation provides a mechanistic link between TDO2 blockade and reduced fibroid growth and ECM accumulation.

Another gene inhibited by 680C91 was CBX4, which is part of the PRC1 complex, a multiunit epigenetic regulator involved in silencing of genes [44]. It also has SUMOE3 ligase activity, whereby adding SUMO to proteins it can alter their stability and activity [44–46]. It has a dual role in cancer, acting as a suppressor of T cell antitumor activity and as an oncogene promoting lung cancer cell proliferation [47–49] and angiogenesis in liver cancer [50]. We previously demonstrated that CBX4 and its associated super-enhancer lncRNA RP1-353N14.12 are up-regulated in fibroids, particularly in MED12-mutated tumors and tumors from Black patients [21]. Suppression of CBX4 by TDO2 inhibition is therefore likely to exert broad epigenetic effects that contribute to transcriptomic reprogramming in fibroids.

Unexpectedly, we observed elevated VDR expression in fibroids relative to matched myometrium, particularly in MED12-mutated tumors, with suppression of its expression following TDO2 inhibition. These results were unexpected, as prior studies using IHC showed lower nuclear VDR expression in fibroids [51] and in cultured fibroid cells by western blot analysis [52]. Furthermore, patients with fibroids have lower circulating vitamin D levels [53–55], and treatment of fibroid cells with Vit D3 inhibited the expression of MMP2 and MMP9 [56], and decreased ERα, PR-A, and PR-B [57], as well as Wnt/β-catenin and mTOR signaling [58]. VDR expression is lower in many types of aggressive cancers [59,60], but in certain tumors, such as prostate cancer, high VDR is associated with reduced lethality [61]. Similarly, in breast cancer, higher expression of VDR was associated with more favorable characteristics [62]. Cancer studies show that VDR acts as a tumor suppressor by inhibiting cell proliferation, inducing apoptosis, and reducing angiogenesis [63]. This discrepancy may be attributable to differences in experimental systems, detection methods, or the specific cellular compartments analyzed. Notably, our analysis did not distinguish between nuclear and cytoplasmic VDR expression, which may be an important factor given that VDR localization can influence its transcriptional activity. This could be significant because, as previously reported in uterine cancer, displacement of VDR to the cytoplasm is associated with lower grade of endometrial cancer [62]. While VDR has been reported to exert anti-proliferative and anti-fibrotic effects in various tissues, the functional significance of its increased expression in fibroids remains unclear. It is possible that elevated VDR expression reflects a compensatory or context-dependent response; however, this hypothesis requires further experimental validation. Future studies examining VDR localization, activity, and downstream signaling will be necessary to clarify its role in fibroid biology.

TDO2 inhibition also altered multiple non-coding RNAs, including lncRNAs and miRNAs, all of which exhibited expression patterns opposite to those observed in fibroid tissues. The more pronounced differential expression of these transcripts in MED12-mutated fibroids further supports their functional significance in fibroid pathogenesis. Several validated lncRNAs have established oncogenic roles. LINC02568 has been shown to promote breast progression and regulate ERα-induced gene transcription by sponging miR-1233-5p and stabilizing ESR1 mRNA [64]. LINC02544 was also overexpressed in breast cancer. The silencing of this lncRNA reduced CARPIN expression and up-regulated miR-497-5p, thereby inhibiting triple-negative breast cancer cell proliferation [65]. LINC02544 is also up-regulated in lung cancer, whereby sponging miR-138-5p regulates like E2F3, thereby influencing cell proliferation, invasion, and metastasis [66]. There are currently no reports on the function of lncRNAs LINC01282 and LINC01310, which we validated as being inhibited by 680C91. MiR-584-5p, which was induced by 680C91 in the xenografts and explant cultures, plays important roles in cancer development and progression, with underexpression in some cancers where it acts as a tumor suppressor, while it is overexpressed in others, acting as an oncogene [67]. Of interest is a report in which miR-584-5p regulated the migration and invasion of non-small cell lung cancer cells through regulation of MMP14 [68]. This raises the possibility that MMP14 could also be a target of miR-584-5p in fibroids, as evidenced by their reciprocal pattern of expression in fibroids. Inhibition of TDO2 by increasing the expression of miR-584 would cause reduced expression of its target MMP14 as shown by our data. Although the specific interactions between the lncRNAs identified in the present study and miR-584-5p remain to be determined, it is plausible that similar regulatory networks exist in fibroids. In addition, several of the differentially expressed protein-coding genes identified in the present study, including MMP11, MMP14, COL11A1, and CBX4, are known to be regulated by miRNAs in other biological contexts, raising the possibility that TDO2 inhibition may modulate these targets through miRNA-dependent mechanisms. Future studies integrating miRNA target prediction and functional validation will be required to determine whether the lncRNAs identified here act upstream of miR-584-5p or other miRNAs to regulate these downstream effectors.

The dysregulation of TDO2 expression in various types of cancer has led to multiple preclinical studies targeting its inhibition [69]. The inhibition of TDO2 results in decreased KYN production, which is an endogenous ligand of the AhR pathway [70], and thus many effects of the TDO2 inhibitors are mediated by the AhR pathway, as demonstrated in our previous studies wherein 680C91 significantly reduced KYN production and suppressed AhR signaling, as evidenced by decreased CYP1B1 expression [11]. In addition, pharmacological inhibition of AhR recapitulated the effects of TDO2 blockade in fibroid explants, including TGF-β3, FN1, CDK2, E2F1, IL-8, and SPARC, supporting a central role for the KYN–AhR axis [11]. The transcriptomic alterations observed here are therefore likely to reflect downstream consequences of this pathway. Specific TDO2 inhibitors have also been tested in multiple preclinical studies in cancer. Administration of 680C91 to mice increased T cell activity, improved dendritic cell function, and reduced metastasis of lung cancer [71]. Treatment of mice with LM-10, another specific inhibitor of TDO2, restored the ability of mice to reject TDO2-expressing tumors [72]. Currently there are no clinical trials with specific TDO2 inhibitors for any disease, and, with the exception of our study, their transcriptomic effects have not been examined. One reason for lack of studies with the specific TDO2 inhibitor 680C91 is due to reported limitations associated with its solubility; however, in our preclinical study, the right conditions for solubilizing this compound were reported [11]. There are several ongoing early clinical trials in cancer with dual TDO2/IDO1 inhibitors [73].

In summary, inhibition of TDO2 produces broad transcriptomic reprogramming in fibroids, affecting both protein-coding and non-coding RNAs. These changes converge on pathways governing ECM production, fibroblast activation, and PI3K/AKT signaling, resulting in reduced fibrotic signaling and tumor regression. While these findings provide important mechanistic insights into fibroid pathogenesis, further studies evaluating pharmacokinetics, safety, and efficacy in clinically relevant settings are needed to assess the translational potential of TDO2 inhibition. Collectively, these findings strongly support that TDO2 inhibition may represent a potential therapeutic approach for uterine fibroids that warrants further preclinical and translational investigation.

## Clinical perspectives

- Background: Uterine fibroids are highly prevalent benign tumors for which current medical treatments are limited by suboptimal efficacy, hormonal side effects, and restricted duration of use. These limitations underscore the need for novel, non-hormonal therapeutic approaches that directly target fibroid pathogenesis.
- Results: In the present study, inhibition of TDO2 resulted in broad transcriptomic reprogramming in fibroid models, including suppression of extracellular matrix-associated and proliferative signaling pathways, inhibition of PI3K/AKT signaling, and reduction of fibrotic and myofibroblast markers. Importantly, the molecular changes induced by TDO2 inhibition were opposite to those observed in fibroid tissue relative to normal myometrium and were more pronounced in MED12-mutated tumors.
- Significance: These findings identify TDO2 as a promising, non-hormonal therapeutic target for uterine fibroids. This preclinical evidence supports continued translational development of TDO2 inhibitors and provides a strong rationale for advancing toward early-phase clinical trials to improve fibroid treatment options and women’s reproductive health.

## Supplementary Material

Supplementary Table S1

## Data Availability

Raw western blot images and associated quantification data supporting the present study have been deposited in Zenodo [74]. RNA-seq data have been deposited in GEO under accession number GSE319228 [75].

## Open Access

Open access for this article was enabled through an open access agreement between the Biochemical Society/Portland Press and the University of California.

## Abbreviations

αSMA: α-smooth muscle actin
AhR: aryl hydrocarbon receptor
ECM: excessive extracellular matrix
LSMCs: fibroid (leiomyoma) smooth muscle cells
FDR: false discovery rate
GEO: Gene Expression Omnibus
GO: Gene Ontology
IDO1: indoleamine 2,3-dioxygenase 1
KEGG: Kyoto Encyclopedia of Genes and Genomes
KYN: kynurenine
lncRNAs: long non-coding RNAs
MSMCs: myometrial smooth muscle cells
NGS: next-generation sequencing
PPI: Protein–protein interaction
siTDO2: siRNA targeting TDO2
sncRNA: small non-coding RNA
Trp: tryptophan
TDO2: tryptophan 2,3-dioxygenase
VIM: vimentin
