# Supplementary material for: In vivo inhibition of TDO2 in fibroids results in widespread alteration in the tumor transcriptome
Source: Clin Sci (Lond). 2026 May 26;140(6):1099–117. doi: 10.1042/CS20260395 (PMC13212361; doi:10.1042/CS20260395)
Supplement: Supplementary Table S1 [file CS-2026-0395_supp.pdf]

Supplementary Table 1: Primer sequences used in the study

Primers	Sequences
FBXW2 (Forward)	5'-CCTCGTCTCTAAACAGTGGAATAA-3'
FBXW2 (Reverse)	5'-GCGTCCTGAACAGAATCATCTA-3'
VDR (Forward)	5'-CCTCCAGTTCGTGTGAATGA-3'
VDR (Reverse)	5'-CGAGTCCATCATGTCTGAAGAG-3'
MMP11 (Forward)	5'-GTGGCAGCCCATGAATTTG-3'
MMP11 (Reverse)	5'-GGGTAGCGAAAGGTGTAGAAG-3'
MMP14 (Forward)	5'-GACTGTCAGGAATGAGGATCTG-3'
MMP14 (Reverse)	5'-CGTGTCCATCCACTGGTAAA-3'
LINC02568 (Forward)	5'-AGCTCCAACTACCGTCTACT-3'
LINC02568 (Reverse)	5'-GAGGGCTGCTTTCTTCTGT-3'
LINC01310 (Forward)	5'-GCCTGGCTTCTAGACACTTC-3'
LINC01310 (Reverse)	5'-CCCGTGATAACCAACACTCTTA-3'
LINC02544 (Forward)	5'-GCTGCTGTCTTTGTGCTTG-3'
LINC02544 (Reverse)	5'-CCCTGGATTCGTGAACAGTAG-3'
LINC02182 (Forward)	5'-GTGTCAGCTGTTCTTCTGTCT-3'
LINC02182 (Reverse)	5'-CAGATTCCATGCTGTGTCCT-3'
TDO2 (Forward)	5'-GCGATCAACTGTGAGTGATAGG-3'
TDO2 (Reverse)	5'-GGTTGGGTTCATCTTCGGTATC-3'
RNU6-2 (Forward)	5'-CGCTTCGGCAGCACATATAC-3'
RNU6-2 (Reverse)	5'-AGG GGCCATGCTAATCTTCT-3'
miR-584-5p (Forward)	5'-AGTTATGGTTTGCCTGGGA-3'
miR-584-5p (Reverse)	5'-GTCCAGTTTTTTTTTTTTTTTCTCAGT-3'
